# Design and physico-mechanical evaluation of fast-dissolving valsartan polymeric drug delivery system by electrospinning method

**DOI:** 10.22038/IJBMS.2021.58713.13041

**Published:** 2021-12

**Authors:** Mohammadreza Mohammadreza, Pariya Iraji, Zahra Mahmoudi, Niloufar Rahiman, Abbas Akhgari

**Affiliations:** 1 Department of Pharmaceutics, School of Pharmacy, Mashhad University of Medical Sciences, Mashhad, Iran; 2 Targeted Drug Delivery Research Center, Pharmaceutical Technology Institute, Mashhad University of Medical Sciences, Mashhad, Iran; 3 Nanotechnology Research Center, Pharmaceutical Technology Institute, Mashhad University of Medical Sciences, Mashhad, Iran; 4 Department of Pharmaceutical Nanotechnology, School of Pharmacy, Mashhad University of Medical Sciences, Mashhad, Iran; # Equally Contributed as First Author

**Keywords:** Hypertension, Nanofiber, Polymers, Polyvinylpyrrolidone, Valsartan

## Abstract

**Objective(s):**

Chronic hypertension is a pervasive morbidity and the leading risk factor for cardiovascular diseases. Valsartan, as an antihypertensive drug, has low solubility and bioavailability. The application of orodispersible films of valsartan is suggested to improve its bioavailability. With this dosage form, the drug dissolves rapidly in saliva and is absorbed readily without the need for water.

**Materials and Methods:**

For this purpose, valsartan with polyvinylpyrrolidone (PVPK90) polymer were exposed to the electrospinning technique to construct orodispersible nanofilms. The optimum obtained nanofiber, selected by Design-Expert software, was evaluated in terms of mechanical strength for evaluation of the flexibility and fragility of the nanofibers. The drug content, wettability, and disintegration tests, as well as the release assessment of the nanofibers, were performed followed by DSC, FTIR, and XRD assays.

**Results:**

The uniform nanofibers’ diameter increased with the increase of the polymer concentration. The tensile test verified a stress reduction at the yield point as the polymer concentration increased. Then, the 492 nm nanofiber with above 90% drug encapsulation, containing 8% polymer and 18% valsartan made below 9 kV, was selected. The wetting time was less than 30 sec and over 90% of the drug was released in less than 2 min. The XRD and DSC studies also confirmed higher valsartan solubility due to the construction alternations in nanofibers. The FTIR examination indicated the chemical bonding between the drug and the polymer.

**Conclusion:**

The selected nanofibers of valsartan present the essential drug feature and acceptable drug release for further investigations.

## Introduction

Today, high blood pressure is one of the most common chronic diseases of the middle-aged population that can only be controlled, not completely treated. To control blood pressure, various medications, including diuretics, beta-blockers, angiotensin-converting enzyme inhibitors (ACEIs), calcium channel blockers (CCBs), and angiotensin receptor blockers (ARBs) may be used (1–3). Valsartan is one of the FDA-approved drugs for controlling blood pressure by blocking the angiotensin II receptor (4, 5). Mechanistically, angiotensin I is converted into angiotensin II by the angiotensin-converting enzyme kinase II. Angiotensin II is the main factor of hypertension in the renin-angiotensin system. The mechanism of angiotensin II in inducing hypertension is related to vasoconstriction, stimulation of production and releasing of aldosterone, cardiac stimulation, and renal sodium reabsorption. Valsartan specifically blocks Angiotensin I receptor (AT1) in some tissues, such as vascular smooth muscle and adrenal glands. It also inhibits vasoconstriction and aldosterone secretion (6, 7). This drug is rapidly absorbed in oral dosage forms and its bioavailability is 25% (8, 9). The protein binding of this drug is 95% and generally excretes intact through biliary excretion (10, 11). Valsartan dissolution is pH-dependent (8, 9) and is a weak acid (pKa= 8.15) that remains non-ionized in the gastric environment. For this reason, it has poor solubility (3.08 μg/ml) but high intestinal absorption (12). On this basis, it has been suggested that adjusting the dissolution medium at a low pH will improve the absorption and bioavailability of valsartan since the bioavailability of the drug is highly dependent on its solubility (13, 14). This drug would be classified in subgroup II of the Biopharmaceutical Classification System (BCS), which indicates low dissolution and high permeability (15).

The oral solid formulations may not meet the needs of some patient populations such as pediatrics, geriatrics, patients with dysphagia, nausea, and vomiting, and who are confined to liquid formulations, as these patients are unable to eat and require oral formulations beyond the standard formulations on the market (16–19). There are great achievements in nanomedicine for the above-mentioned patients. Orodispersible film (ODF) is a type of drug delivery system for local and systemic drug delivery that acts through buccal melting. They are single or multilayer thin films designed for the purpose of rapid hydration and subsequent liberation of the active pharmaceutical ingredients (API) in the mouth through saliva, and formation of a suspension or solution in the saliva without the requirement of water ingestion or mastication (20, 21). The released drug from ODFs may apply its effect in the buccal cavity, directly absorbed through the buccal cavity (transmucosal absorption), or maybe absorbed by the intestine after swallowing the solvated drug in the saliva (intestinal absorption). The percentage of the drug in the film is usually 1–25%. In fact, the lower the therapeutic dose, the faster the drug dissolves in the buccal cavity (22).

ODFs can provide precise and flexible dosing and facilitate personalization of the dosage strength (23), especially for drugs with a narrow therapeutic index, such as warfarin (24). They also support the early onset of action and enhanced bioavailability due to their absorption from the pre-gastric region. This kind of absorption circumvents the hepatic first-pass effect metabolism and is more appropriate for the drugs with hepatic metabolism (25, 26). In this regard, multiple electrospun nanofilms have been constructed as ODFs with promising results, such as aspirin for its less gastric irritation (27) and isoniazid for tuberculosis for more adherence to therapy (23). Ondansetron ODFs are also applicable in the market for patients with vomiting issues who are unable to use edible products (28)ODFs usually result in enhanced bioavailability with faster onset of action. In addition, ODFs are flexible. Unlikely to orally disintegrating tablets, they do not require any special packaging for protection during transportation and storage. The critical quality attributes (CQAs).

By mechanical modification of the nanofibers, they can be converted into elastic and homogenized nanofibers. The mechanical strength of films is essential in preventing their damage and fragility. High surface-to-volume ratio and high tensile strength are the exclusive properties of nanofibers (29). Nanofibers are attractive nanocarriers due to their high surface and interconnected porous structure (30). Contrary to all advantages, these films are fragile due to their high porosity and low mechanical strength. In addition, the resulting disintegrated materials may become aggregated on the tongue which may be unpleasant for patients (31).

A fundamental component of films is a polymer that forms a matrix of the drug. The film’s strength is directly correlated to the nature and quantity of the polymer. The ideal polymers for this purpose should be pure, non-toxic, non-irritant, and have wetting and diffusing ability with enough resistance (32). The other component of films is a solvent for the dissolution of the polymer and making a viscose solution. The API is added to the viscose solution and after their complete integration, it is ready for further processes such as electrospinning (22). 

**Figure 1:**
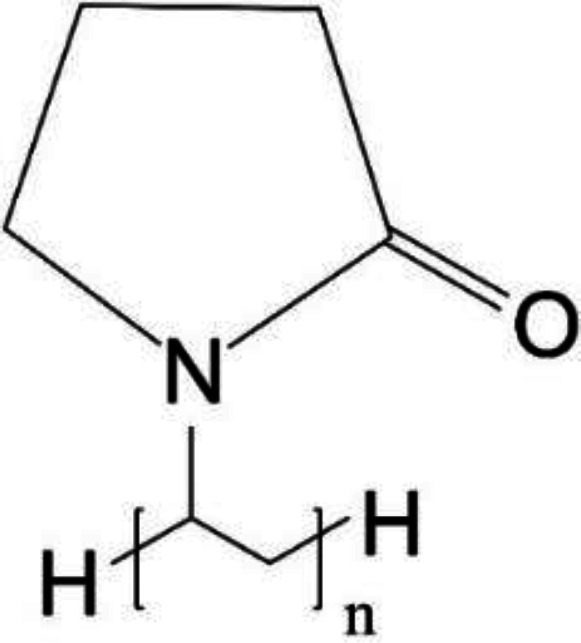
Polyvinylpyrrolidone (PVP) structure and functional groups

Polyvinyl pyrrolidone (PVP) is a synthetic and non-toxic polymer with an amide ring (Figure 1) that enables the formation of hydrogen bonds and makes it highly soluble in water and organic solvents. It can absorb water up to 40% of its weight and organize a gel-like construction (33, 34). PVP has intrinsic adhesive properties and is able to build complexes with different materials which makes this polymer highly applicable in pharmaceutical industries (35).

Electrospinning is one of the best simple and beneficial methods for the production of porous nanofibers (36–38) alongside other methods such as solvent casting, hot-melt extrusion, and printing technologies (20, 35, 39). 

Exposure of the polymer solution to a high voltage results in charged polymeric droplets. Then, they are extruded through a nozzle in the electrospinning machine, and the droplets are elongated by effect of the electrostatic force. The nanofiber is constructed after solvent evaporation from the droplet (40–43).

Valsartan, as a drug for hypertension therapy with low bioavailability and hepatic metabolism, could be a candidate for this purpose. Due to the properties of the drug, the condition for increasing its bioavailability is created by the production of nanofibers through the electrospinning method. In this study, an ODF form of valsartan was formulated to evaluate its physicomechanical properties in this nanoformulation.

## Materials and Methods

***Materials***

Valsartan (Tianyu Pharm, China), PVPK90 (Fluka Chemie AG, Switzerland), Distilled water (Pharmacy school of Mashhad, Iran), KH_2_PO_4_ (Merck, Germany), NaOH (Merck, Germany), HCl 37% (Merck, Germany), and Ethanol 96% (Hamoon Teb Markazi, Iran) were obtained from the indicated sources.

***Experimental design***

The experimental design was applied to determine experimental formulations, the correlation between variables and responses, and the selection of the optimum formulation. For this purpose, the effect of formulation parameters on the characteristics of the resulting nanofibers was assessed. The independent variables studied in this research, according to the preliminary reviews, are the valsartan concentration (6–30 %), PVP concentration (8–14%), and voltage (9–15 kV), which were considered as the effective variables in nanofibers’ characteristics (Table 1).

As a result, according to the Box-Behnken design with 15 axial points and 0 center points, fifteen formulations were selected according to Table 1. Subsequently, the response surface methodology was applied in Design-Expert 10 software and the optimum formulation was selected on its basis after the optimization process by data analysis with ANOVA tests. The effect of drug percentage and voltage on the nanofibers’ diameter, drug, and polymer impact on yield-stress of nanofibers, drug, and polymer impact on the percentage of drug content relative to the theoretical value, and drug and polymer impact on the wettability of the constructed nanofilm were considered in the experimental design.

***Preparation of the spinning solutions***

*The PVPK90 solution was *prepared due to the determined w/v% in ethanol 96° as its appropriate solvent. Separately, valsartan was weighted in a determined mass and added to the above-mentioned solution. The desired volume was reached with ethanol 96°. The solution was subjected to the electrospinning process after 24 hr. The ratio of drug: polymer solution was based on the full factorial design described in section 2.2. 

***Electrospinning process***

The electrospinning device (Uniaxial electrospinning machine, ES1000 model, *Fanavaran* Nano Meghyas Co, Iran) is equipped with a 0-35 kV power supply. The cylinder collector is covered by aluminum foil. Electrospinning solutions containing different ratios of the drug: polymers were loaded in 5 ml syringes. The syringes were placed horizontally on a separate injection pump equipped with digital control. The 20G steel needle tip was used as the nozzle and the horizontal distance of the collector to the needle tip was 150 mm. The feeding rate which was controlled by a syringe pump was fixed at 0.75 ml/hr with the rotational speed of 170–190 rpm. The injection pump rotated on its axis with a rate of 4 mm/min. The process was performed at room temperature (25.0 ± 0.2 °C) with a relative humidity of 45%. Finally, the foil containing the nanofibers was removed from the collector and stored in the refrigerator (4–8 °C) for further studies.

***Experimental***

*Scanning electron microscopy (SEM)*

The purpose of subjecting nanofibers to scanning electron microscopy (SEM) is to assess the diameter of the nanofibers and their uniformity. The morphology of electrospun nanofibers was manifested using a FESEM (VP 1450, ZEISS, US) after atomic bombardment with the mixture of gold and palladium in a vacuum and they were imaged with a magnification of 10000. The mean diameter of the electrospun nanofibers was determined using Image J software on SEM micrographs. In addition, the images were used to investigate the morphology and assess the presence or absence of globules for the selection of the optimum formulation.

*Elasticity and tensile strength tests*

‌Nanofibers with a dimension of 1 cm ×4 cm and determined thickness were cut and attached to the cardboard frames with double-sided glue and placed between the two clips of Hounsfield H50SK device equipped with 5 kN loading cell and the tensile test was performed at the speed of 5 mm/min. Then a nanofiber was gradually stretched and this process continued until the nanofiber broke. The amount of stress against increasing nanofiber’s length was also measured. After recording the corresponding chart, Young’s modulus, yield stress, work of failure, and elongation were investigated as well. This test was carried out in triplicate for each sample for the selection of the best formulation. 

*Determination of drug content*

To determine the drug content in nanofibers, pieces of nanofibers with specific weight were separated from each nanofiber and dissolved in phosphate buffer, and their absorptions were determined at 250 nm (44) by a Unico UV-2100 (Unico, US) spectrophotometer. Since the polymer has no absorption at λ=250 nm, it does not interfere with the determination of drug absorption. The determination of absorption was performed in triplicate for each sample. The average of the three absorptions was calculated and drug percentage was determined according to the standard curve and compared with the theoretical amount (equation (1)).

Drug content (%) = Actual valsartan content in nanofibers / Theoretical valsartan content in nanofibers 100                    (1) 

*Wettability test*

Filter papers with a dimension of 6 cm × 6 cm were selected and wetted with phosphate buffer pH=6.8 for mimicking the oral cavity environment. Nanofibers with a dimension of 2 cm ×2 cm were cut and placed on the wet filter papers, and then the wetting pattern was recorded until the full disappearance of the nanofilm (45). This analysis was performed in triplicate for each sample.

*Disintegration test*

Fibers with a dimension of 2 cm ×2 cm were cut and placed in a beaker with 25 ml phosphate buffer and put on a stirrer with 50 rpm (for simulation of the movement of the tongue or the oral cavity movements) at the temperature of 37 ± 2 °C. The disintegrating time was recorded until the complete dissolution of the nanofiber in the phosphate buffer (pH=6.8) (46). The analysis was performed in triplicate for each sample**.**

*Selection of the optimum formulation*

The results of the performed tests on samples were represented through a mathematical model with Design-Expert software. It demonstrated the relations between variable factors (polymer concentration, drug concentration, and voltage) with nanofiber characteristics like mean nanofiber diameter, percentage of drug content, tensile strength and elasticity of nanofiber, wetting time, and disintegrating time. After several reviews, a nanofiber was selected with the following criteria: nanofiber’s diameter below 1000 nm, yield point above 0.1, drug entrapment in nanofibers above 90%, and wetting time less than 30 sec. The following complimentary tests were performed on the optimum selected formulation (formulation B, according to Table 2).

*Differential scanning calorimetry (DSC)*

The valsartan powder, PVPK90, the physical mixture of drug and PVPK90* (with the selected percentage),* and a selected piece of nanofiber were subjected to DSC, and their respective thermograms were obtained. Three milligrams of each sample were placed in a specific aluminum pan and inserted into the DSC device. Analysis was performed with a DSC machine (model 822ᵉ, Mettler Toledo, Switzerland). The thermal behavior of samples was analyzed in the range of 35–350 °C at a speed of 10 °C/min under nitrogen gas flow and recorded against a vacant aluminum pan as reference.

*Fourier transform infrared spectroscopy (FTIR)*

The FTIR test was performed to study the functional groups and possible chemical bonds and physical interferences between the drug and the polymer in the electrospun nanofibers. The analysis was performed on drug powder, polymer powder, physical mixture of drug and polymer in the selected formulation and the selected nanofiber. Three milligrams of each sample were inserted into the FTIR spectrophotometer (Spectrum Two model, Perkin Elmer, USA) separately with a scanning range of 400 to 4000 cm ^-1^ at a resolution of 8 cm ^-1^. The resulted data were presented and recorded by PerkinElmer Spectrum Version 10.03.02 software.

*X-ray diffraction (* *XRD)*

The XRD analysis was carried out for valsartan powder, PVPK90 powder, the physical mixture of drug and PVPK90 powder, and a selected piece of produced nanofiber. In the XRD apparatus (PW3710 model, Philips Analytical, the Netherlands), radiation was created by copper as an anode. The procedure was carried out in the 2θ interval and 0 °C, 40 kV and 30 mA. The source of the raw data was PHILIPS-binary (scan)(.RD). The results and peaks were investigated and recorded.

*Dissolution study and drug release*

For release test conduction, 10 mg of the optimum nanofiber, containing 0.0144 mg of valsartan, was weighed and dissolved in 100 ml of phosphate buffer (pH=6.8) (in the concentration range of its calibration curve) and incubated at 37 °C on a stirrer with the rate of 50 rpm (24). The solution temperature was monitored by a thermometer until the end of the procedure. Sampling with micropipette was performed at 30-second intervals and the sampling volume was replaced with an equal volume of buffer, for maintaining the sink condition during the *in vitro* release study (47). This procedure was performed in 4 min and then the samples were placed in the spectrophotometer (Unico UV-2100, Unico, US) and their absorptions were read at 250 nm. The absorptions were converted to concentration with the standard equation and the release percentage of the drug was calculated at different time points. The dissolution rate of an equivalent amount of valsartan and physical mixture of polymer and valsartan were also assessed by this procedure to achieve a comparable rate of dissolution. These experiments were performed in triplicate for each sample and the mean cumulative release was plotted. 

***Statistical analysis of data***

Linear regression and analysis of variance were used to evaluate the effects of the variable factors on each experimental response and the interactions between them. For this purpose, Design-Expert software was used and polynomial mathematical models were obtained. The final coefficients in this model were statistically significant. According to these mathematical models, three-dimensional charts were created in order to demonstrate the influence of variable factors on responses.

## Results

***Fibers morphology***

**Figure 2:**
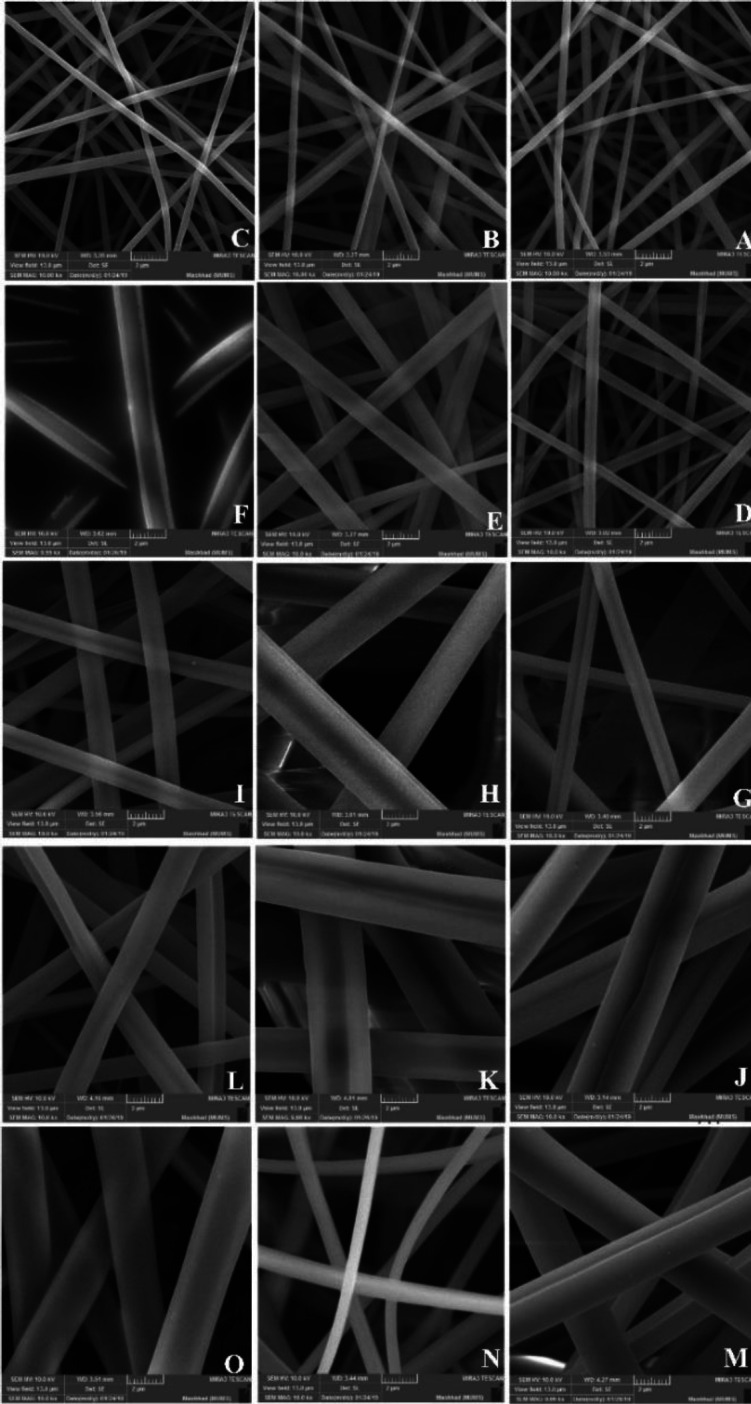
Scanning electron microscopy (SEM) images for the fifteen formulations constructed with components according to Table 1 (scale bars 2 μm and magnification of 10000)

The SEM images of nanofibers are demonstrated in Figure 2 and their mean diameters are shown in Table 2. Nanofibers were assessed in terms of homogeneity and diameter. Images were prepared with a magnification of 10000 by Image J 1/50i software. The *coefficient of determination *was equivalent to 0.7. 

**Figure 3:**
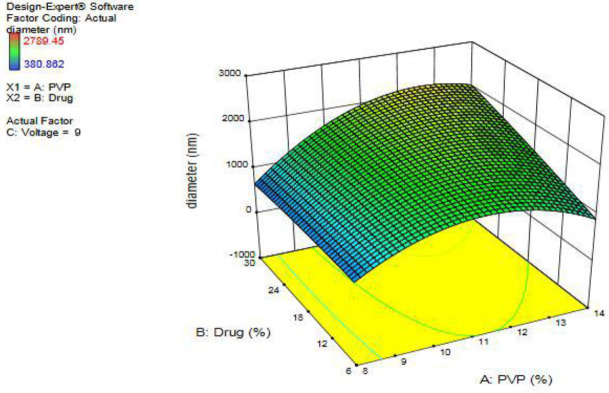
Three-dimensional chart of the effect of drug percentage and voltage on the nanofibers’ diameter

The results are shown in three-dimensional Figure 3. The area-response diagram was obtained by the mathematical equation (2): 

16785.6 + (1396.38 × PVP) + (95.4734 × Drug) + (1584.33 × Voltage) + (7.96854 × PVP × Drug) +(16.2228 × PVP × Voltage) + (-12.607 × Drug × Voltage) + (-72.9232 × PVP^2^) + (-0.257935 × Drug^2^) + (-66.5749 × Voltage^2^)                     (2) 

***Tensile strength test***

**Figure 4:**
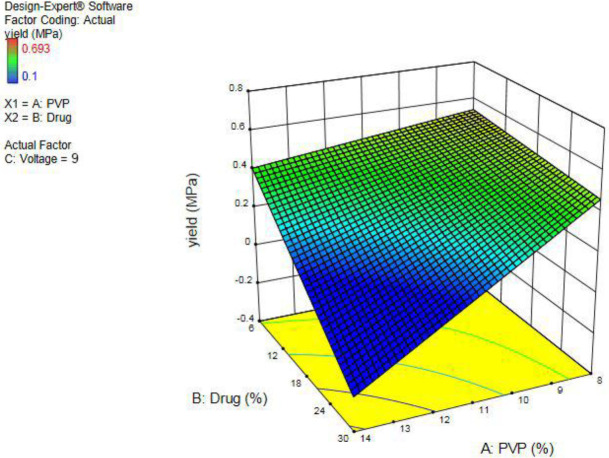
Three-dimensional chart of drug and polymer impact on yield-stress of nanofibers

The result of the tensile strength test was evaluated and recorded according to the strain-stress diagram and Young’s modulus. The resulted data of Young’s modulus mean, yield stress, elongation, and work of failure are shown in Table 2. The three-dimensional chart of drug and polymer impact on the yield-stress of nanofibers is shown in Figure 4. The *coefficient of determination* is equal to 0.735, and the diagram has been obtained by the mathematical equation (3):

-0.177141 + (-0.0420783 × PVP) + (0.0893027 × Drug) + (0.0609287 × Voltage) + (-0.00393741 × PVP × Drug) + (0.00364704 × PVP × Voltage) + (-0.00508963 × Drug × Voltage)                     (3)

***Drug content test***

**Figure 5:**
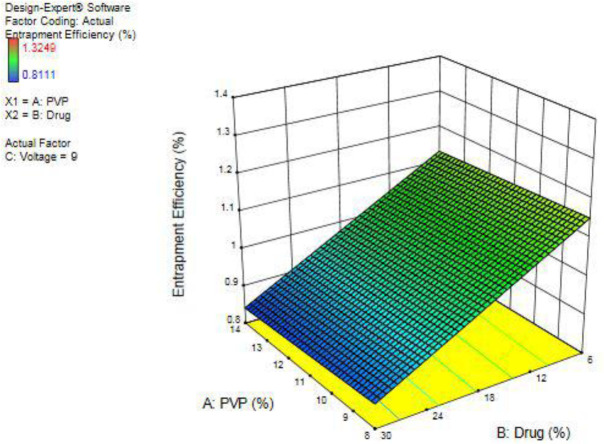
Three-dimensional chart of drug and polymer impact on the percentage of drug content relative to the theoretical value

The amount of drug in samples in relation to the theoretical amount was evaluated and recorded. The relation between variable items and drug content is shown in the three-dimensional diagram in Figure 5. The *coefficient of determination* is equal to 0.697, and the diagram was obtained by the mathematical equation (4):

1.14901 + (-0.00339444 × PVP) + (-0.0122343 × Drug) + (0.00908175 × Voltage)                     (4)

***The wettability and disintegration test***

The results of these two tests are shown in Table 2. The wetting pattern starts from all edges of the nanofiber and continues until it completely disappears, as demonstrated in Figure 7.

**Figure 6:**
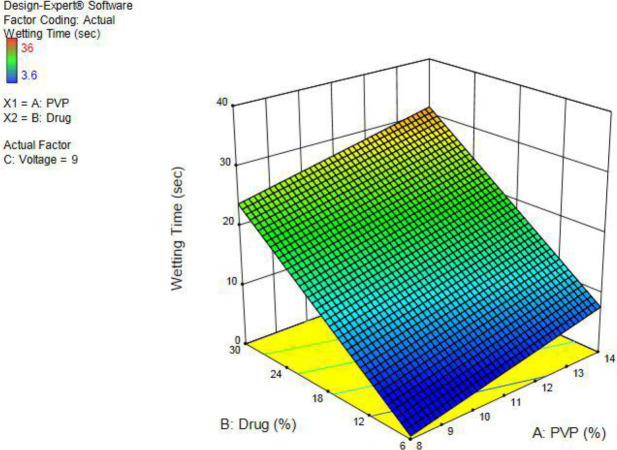
Three-dimensional chart of drug and polymer impact on the wettability of the constructed nanofilm

Disintegration occurs by exposure of the film to the phosphate buffer solution. Initially, the film becomes gel-like by absorption of the buffer and then proceeds to complete dissolution according to Figure 8. The relation between variables and wetting time was evaluated by Design-Expert software in the form of a three-dimensional diagram. The *coefficient of determination* is equal to 0.832, and the diagram was obtained (Figure 6) by the mathematical equation (5):

-3.65624 + (1.23185 × PVP) + (0.985582 × Drug) + (-0.965291 × Voltage)                     (5)

***Selection of the optimum formulation***

After evaluations on the results of the Design-Expert software, and according to the criteria of selection of the appropriate nanofiber (optimal size, drug encapsulation efficiency, and Young’s modulus, yield stress, elongation percentage, wetting time, and disintegration time) which were previously indicated, finally, the optimum nanofiber was selected. This selected nanofiber contains 8% polymer, 18% valsartan and was produced at a voltage of 9 kV.

***Morphology of the optimum formulation***

The formulation B in Figure 2 demonstrates the SEM image of the selected nanofiber, in which the diameter of the nanofibers was measured with Image J 1/50i and the mean size of this nanofiber was equivalent to 492.6±4.5 nm. The ANOVA results for the average diameter of nanofibers were shown in Table 3.

***Tensile tests***

The results of the tensile test and the strain-stress diagram are shown in Figure 9, which indicate and confirm Young’s modulus and the yield point for the optimum formulation of valsartan nanofiber. The ANOVA results for average Young’s modulus of nanofibers were shown in Table 4.

***Drug content***

The percentage of drug content in relation to the theoretical amount was performed in triplicate and was equivalent to 96.5±3.1% for the optimum formulation. The ANOVA results for average drug content in the nanofibers were shown in Table 5.

***Wettability and disintegration time***

According to Table 2, the wettability and disintegration time for the optimum formulation (formulation B) were equivalent to 7.3±0.2 and 66.5±0.6 sec, respectively. The disintegration was measured through the interaction of ODF with media and the slow disintegration of its edges and corners and becoming porous. Eventually, the film disintegrates completely with small scattering particles (48). The ANOVA tables for average wetting and disintegration times of nanofibers were shown in Table 6 and Table 7.

***DSC analysis***

The thermal behavior of valsartan, PVPK90, physical mixture of drug and nanofiber, and nanofiber were evaluated and recorded. The investigations in diagrams demonstrate that the melting point of valsartan was eliminated in the final nanofiber, which can be an indicator of chemical alternations. The results of the XRD can confirm the results of the DSC analysis (Figure 10).

***FTIR analysis***

Valsartan showed a peak in 1730.7 cm^-1^, which is related to the acidic group of valsartan. This peak is also evident in the physical mixture of drug and polymer. This peak has been eliminated in the selected nanofiber, which shows the chemical reactions and appears to be the result of the reaction of the valsartan acidic group with the amide group of PVP (Figure 11).

***XRD***

The XRD pattern of valsartan (Figure 12) shows its semi-crystalline features. The physical mixture of valsartan with PVP has caused a descent in the related peak, showing the enhancement of valsartan dissolution. The optimum nanofiber of valsartan also shows this pattern of semi-crystallinity with a single sharp peak that could be due to the nanofibers’ formation.

***Dissolution study and drug release from the selected nanofiber***

The results of the drug release shown in Figure 13 indicate that about 90% of valsartan was released from the nanofiber in less than 2 min. Intact valsartan was dissolved about 38% in the respective medium after 4 min. During this time, in the physical mixture of valsartan and PVP, valsartan demonstrated a 70% dissolution rate. 

## Discussion

 In order to attain an optimum formulation for the production of valsartan nanofibers with PVP as a polymer by electrospinning method, Design-Expert software was used for the experimental design. After evaluating the parameters’ variations, the three parameters of drug concentration, polymer concentration, and voltage were eventually determined, while other parameters were kept constant. By evaluating the variations in the variables, the experimental design determined fifteen formulations. By evaluating SEM images, it was validated that nanofibers lack beads, and it appeared that the drug was well localized among the polymer particles with a homogeneous appearance. Except for seven formulations, other formulations were in the nanometer range and as polymer concentration increased, the nanofibers’ diameter increased too. By evaluating the samples’ diameters, it was found that the narrowest nanofibers belonged to the nanofibers with a concentration of 8% of the polymer. By increasing the polymer concentration, the polymer mass in the spinning jet increased and by induction of disturbance in the fluid flow, it formed nanofibers with higher diameters (49, 50).

This event was proven in a study in which increasing the polymer concentration had a significant influence on the morphology of the nanofibers. As polymer concentration increases, the diameter of the nanofibers increases too, which may lead to the formation of microfibers (51). The evaluation of the tensile test determined that by increasing tension at the yield point, nanofibers’ resistance against tension increased. By increasing polymer concentration, yield stress decreased and nanofibers would be less flexible against tension, according to the tensile test results. The results showed that the maximum tension in the yield point was related to the nanofibers with a concentration of 8% of the polymer. ODFs should be flexible enough during the production process. In addition, they should not be too fragile which impedes the precise cutting of the film. The films that are flexible enough may be stretched during cutting. Since drug dosing depends on the film’s surface, flexibility ensures accurate dosing. According to the literature, there are no specified ranges for tensile strength in ODFs, and this parameter can only be used to compare samples to select the best formulation (52).

The evaluation of the drug content test confirmed that the most drug content ratio in relation to theoretical amount is demonstrated in formulations with the concentration of 6% valsartan‌‌‌, while the least amount of this ratio is demonstrated in the formulations with the concentration of 30% valsartan. It appears that in low concentrations of valsartan, the drug could be properly placed in the polymer structure, and drug entrapment efficiency increases. High entrapment efficiency has been shown to have a great impact on the prevention of drug loss during the electrospinning process (53).

The evaluation of the wetting and disintegration time showed that by increasing drug concentration, wetting time increased. Since valsartan is poorly water-soluble, increasing its concentration increases the wetting time. On the contrary side, in one study, the preparation of nanofibers with water-soluble polymer helped the dissolution of the drug and reduced the disintegration time of the film (54). Saliva penetrates the porous spaces of the ODFs and the film starts to become wet. The porosity increases at lower voltages, and the wetting time decreases as the polymer concentration increases. PVP performs as a water-soluble substance and causes saliva to penetrate through the nanofibers and form a gel-like structure. 

The IR spectrum for PVP shows a peak at 2920.9 cm^-1^, which is related to the amide group of PVP which has also been validated by another study (55). The IR analysis for valsartan validates a peak in 1604 cm^-1 ^which is expressive of C=O in the amide group. A second peak is observed in 1730.7 cm^-1^ which is expressive of C=O vibrations in the carboxylic acid group. This IR peak for valsartan was also confirmed by another study (56). This peak has also been detected in the FTIR spectrum of the physical mixture of drug and polymer, but in the optimum nanofiber, it appears that the peak related to the carbonyl group of carboxylic acid has been removed and the peak of the carbonyl of the amide group has shifted to left, which is indicative of the occurrence of a chemical reaction. The chemical bonding is usually hydrogen bonding or some electrostatic bonds that normally have no adverse effects on the biological activity of the drug and are reversible. It is notable that there is a probability for the formation of hydrogen bonds between valsartan and PVP, as valsartan could be a hydrogen bond donor due to the presence of OH in its structure and also a hydrogen bond acceptor due to the presence of carbonyl groups and also because of the unpaired electrons of nitrogen. Thus, OH in valsartan can form hydrogen bonds with carbonyl groups or with nitrogen, if its spatial angle in the PVP structure is appropriate. Since PVP is a polymer and has lots of carbonyl groups and also an amide group, it could theoretically form hydrogen bonds with OH in valsartan.

The DSC analysis determines a peak of valsartan at 101 °C which is indicative of its melting point and was also validated in other studies (56,57). A peak of 75 °C is also demonstrated for PVP, which appears to be due to water loss. The presence of water in PVP performs like a plasticizer and shifts the melting point of the polymer to the left side. The peak of polymer indicating its melting is covered by the water evaporation peak. In one study on carbamazepine and PVP as a polymer, the DSC analysis of polymer demonstrated a peak in the range of 75–140 °C and explained that due to the moisture absorption of polymer, the peak is related to the water loss (58). In the physical mixture of polymer and drug, both their peaks are demonstrated on the diagram but the drug peak is not demonstrated completely due to its overlap with the peak of polymer. The DSC diagram of the nanofiber shows that the peak is not demonstrated at 101 °C which could suggest the construction alternations after the formation of nanofiber. 

The XRD pattern of valsartan (Figure 12) shows its semi-crystalline characteristics notably due to the broad peak at 22° in 2θ diffraction that could be a mixture of amorphous and crystalline phases, indicated by the two hump-shaped peaks (56). The physical mixture of valsartan with PVP has caused a descent of the mentioned peak at 22° validating the effect of PVP in the enhancement of valsartan dissolution even in a physical mixture with a hydrophilic polymer. The optimum nanofiber of valsartan also shows this pattern of semi-crystallinity with the same peak at 22° in 2θ diffraction with different scale and also a sharp peak at 2θ diffraction angle of 45° which has not been presented in the XRD pattern of valsartan, PVP, or their physical mixture. This phenomenon may be due to nano-sized fibers giving rise to the broadened diffraction peaks usually at relatively high angles.

The results of the release test show that 90% of the drug is released in less than two minutes. Several factors, such as the diameter and porosity of nanofibers play important roles in the release of the drug from the film surface. In low diameters of nanofibers, the release rate increases due to the higher contact surface. In contrast, the higher porosity in nanofibers, increases the release rate of the drug, so that thick nanofibers with high porosity have a higher release rate of the drug than thin nanofibers with low porosity (59). The low voltage applied to the selected nanofiber can play an important role in increasing its porosity. The hydrophilicity of PVP also aids for more quick penetration of the dissolution medium between the nanofiber pores. The intact drug as it has been mentioned in the manuscript is poorly soluble in aqueous media and its dissolution rate is about 38% at pH=6.8 during 4 min. PVP as a water-soluble polymer helped the dissolution of valsartan in aqueous media even in a physical mixture with this drug and enhances its dissolution rate to about 70% in the same condition. As the result, by the formation of a nanofiber between valsartan and PVP, PVP contributes to better dissolution of the drug following its enhanced release (60).

**Table 1:** Percentage of drug, polymer, and applied voltage in nanofiber production

Samples	PVPK90%	Drug%	Voltage (kV)
A	8	6	12
B	8	18	9
C	8	18	15
D	8	30	12
E	11	6	9
F	11	6	15
G	11	18	9
H	11	18	15
I	11	18	12
J	11	30	9
K	11	30	15
L	14	6	12
M	14	18	9
N	14	18	15
O	14	30	12

**Table 2:** Results of nanofibers' diameter according to scanning electron microscopy (SEM) images, mechanical tests on nanofibers, drug content, wetting time, and disintegration time of nanofibers (n=3)

**Formulation**	**Fibers diameter (nm)**	**Sample thickness (µm)**	**Young’s modulus** **(MPa)**	**Yield stress** **(MPa)**	**Elongation** **(%)**	**Drug content ratio to theoretical content (%)**	**Wetting time (sec)**	**Disintegration time (sec)**
A	430.3±11.7	0.21±0.02	3.22±0.21	0.53±0.02	9.5±0.28	100±5.9	11.4±0.6	104.5±2
B	492.6±4.5	0.19±0.01	14.78±0.44	0.69±0.03	7.2±0.35	96.5±3.1	7.3±0.2	66.5±0.6
C	380.8±6.6	0.19±0.009	39.09±1.51	0.54±0.03	2.5±0.28	100±0.6	8.1±0.3	74.5±1.4
D	464.4±9.7	0.19±0.008	22.51±0.97	0.42±0.02	4.9±0.16	81.1±0.004	27.6±3.1	169±3.6
E	941.1±10.2	0.2±0.02	18.66±0.52	0.1±0.02	6.6±0.12	100±7.9	12±3.4	97±3.4
F	925.4±11.1	0.19±0.004	4.65±0.19	0.65±0.08	2.2±0.28	100±15	4±1.03	125±2
G	1452.4±18.3	0.18±0.007	17.5±0.28	0.38±0.03	9.4±0.61	100±0.4	15.6±1.3	142.5±1.5
H	2789.4±36	0.18±0.012	45.06±0.83	0.31±0.02	2.2±0.57	100±0.07	17.3±0.9	156.5±0.7
I	1179.3±34	0.19±0.004	20.6±0.91	0.21±0.03	2.8±0.3	96.5±3.6	11.6±0.6	100±2
J	2323.8±51	0.21±0.02	9.64±0.14	0.13±0.03	2.2±0.66	90.3±0.01	36±3.03	115±3
K	2264.3±36	-	-	-	-	-	-	-
L	1189.0±41	0.18±0.005	33.97±3.1	0.45±0.01	2±0.21	99.2±0.004	6±0.3	55±3.4
M	1145.3±23	0.2±0.015	15.43±0.84	0.16±0.01	1.5±0.16	98.2±0.02	20±1.7	114.5±3
N	902.5±7.8	-	-	-	-	-	-	-
O	2370.6±8.7	-	-	-	-	-	-	-

Performance of the mechanical assays and evaluation of wettability and disintegration tests were not possible due to the ultra-thickness for samples K, N, and O

**Figure 7:**
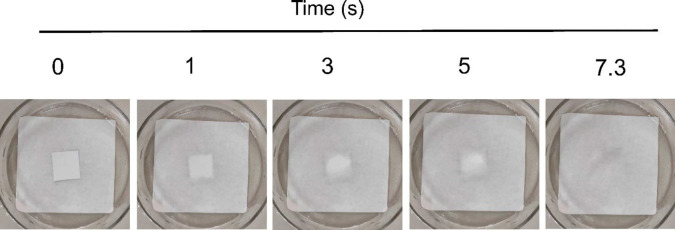
Process of wettability of the nanofiber with 8% polymer and 18% valsartan produced with 9 kV voltage on the filter paper wetted with phosphate buffer at pH=6.8 (n=3)

**Figure 8:**
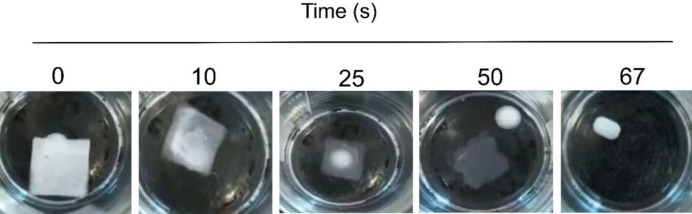
Disintegration process in the nanofiber with 8% polymer and 18% valsartan produced with 9 kV voltage on the filter paper wetted with phosphate buffer pH=6.8 (n=3)

**Table 3:** ANOVA table for the average diameter of nanofibers

**Source**	**Sum of square**	**DOF**	**Mean square**	**F-value**	***P*** **-value**	
	26373083	14	1883792	2958	<0.0001	Significant ****
Residual (within formulations)	19105	30	636.8			
Total	26392188	44				

Significant at the 5% level (*P*<0.05); DOF: Degrees of freedom

**Figure 9:**
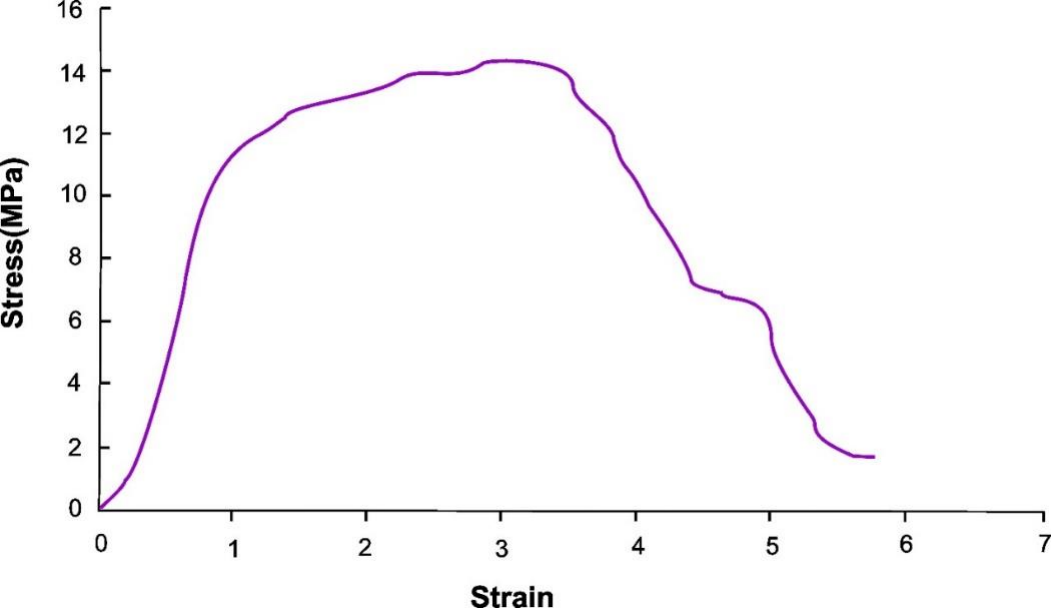
Strain-stress diagram of the nanofiber with 8% polymer and 18% valsartan, produced at the voltage of 9 kV (n=3)

**Figure 10:**
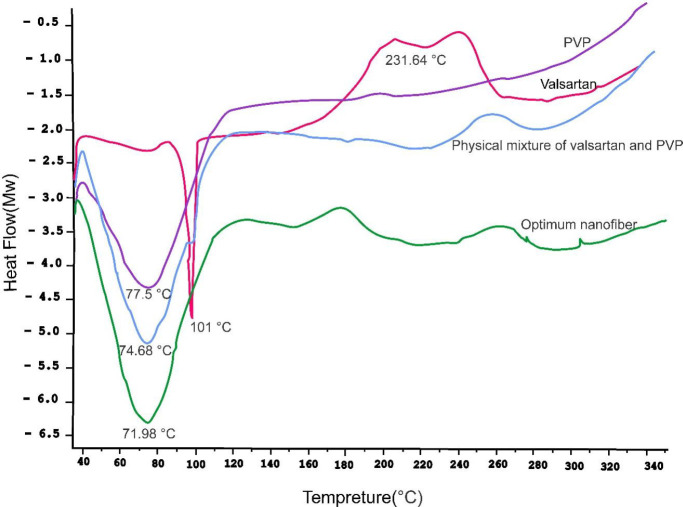
Diagram of the 2.5.7. differential scanning calorimetry (DSC) analysis of valsartan, polyvinylpyrrolidone (PVP), physical mixture of valsartan and PVP, and the optimum selected nanofiber (B)

**Table 4:** ANOVA table for average Young’s modulus of nanofibers

**Source**	**Sum of square **	**DOF**	**Mean square**	**F-value**	***P*** **-Value**	
	5617	11	510.7	393.5	<0.0001	Significant ****
Residual (within formulations)	31.14	24	1.298			
Total	5648	35				

Significant at the 5% level (*P*<0.05); DOF: Degrees of freedom

**Table 5:** ANOVA table for average drug content of nanofibers

**Source**	**Sum of square **	**DOF**	**Mean square**	**F-value**	***P*** **-Value**	
	1074	11	97.66	3.394	0.0060	Significant **
Residual (within formulations)	690.6	24	28.78			
Total	1765	35				

Significant at the 5% level (*P*<0.05); DOF: Degrees of freedom

**Table 6:** ANOVA table for average wetting time of nanofibers

**Source**	**Sum of square **	**DOF**	**Mean square**	**F-value**	***P*** **-Value**	
	2902	11	263.9	103.7	P<0.0001	Significant ****
Residual (within formulations)	61.08	24	2.545			
Total	2963	35				

Significant at the 5% level (*P*<0.05); DOF: Degrees of freedom

**Table 7:** ANOVA table for average disintegration time of nanofibers

**Source**	**Sum of square **	**DOF**	**Mean square**	**F-value**	***P*** **-Value**	
	40340	11	3667	618.6	P<0.0001	Significant ****
Residual (within formulations)	142.3	24	5.928			
Total	40482	35				

DOF: Degrees of freedom

**Figure 11:**
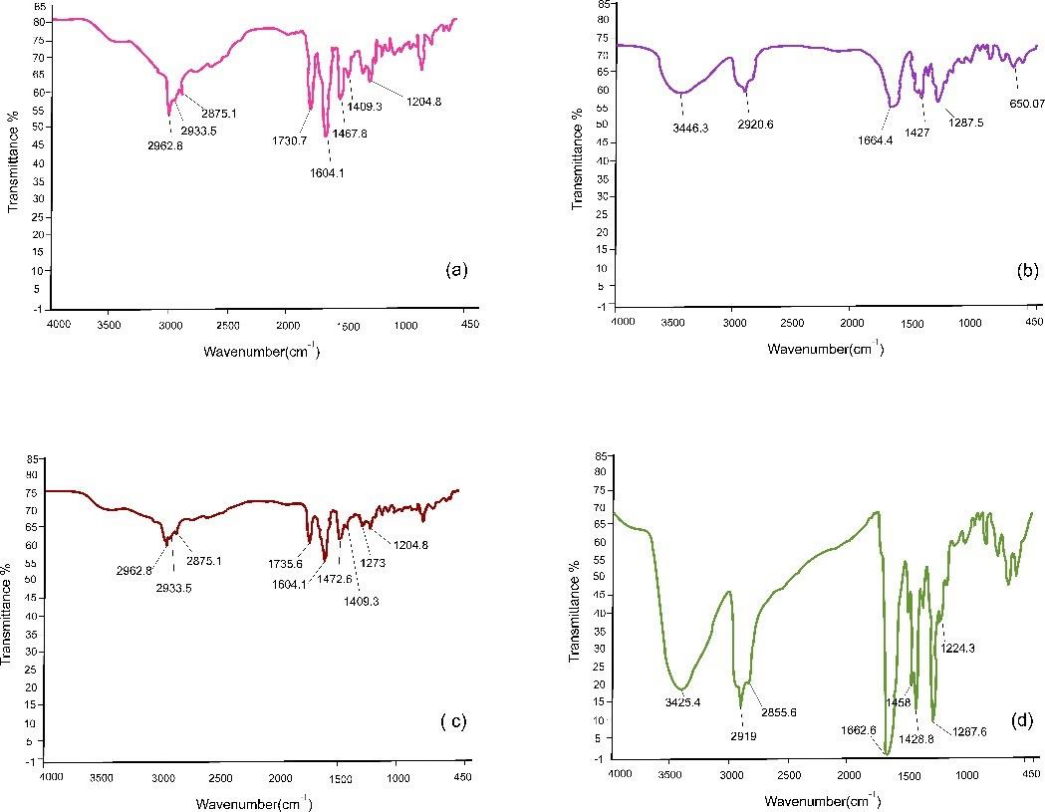
Diagram of the 2.5.8. fourier transform infrared spectroscopy (FTIR) analysis of (a) valsartan, (b) polyvinylpyrrolidone (PVP), (c) Physical mixture of valsartan and PVP, and (d) the optimum selected nanofiber (B)

**Figure 12:**
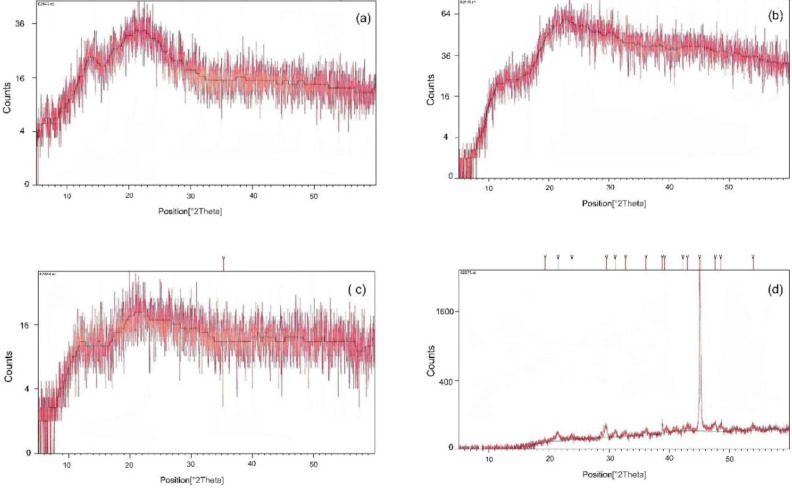
Diagram of the 2.5.9. X-ray diffraction (XRD) analysis of (a) valsartan, (b) polyvinylpyrrolidone (PVP), (c) physical mixture of valsartan and PVP, and (d) the optimum selected nanofiber (B)

**Figure 13:**
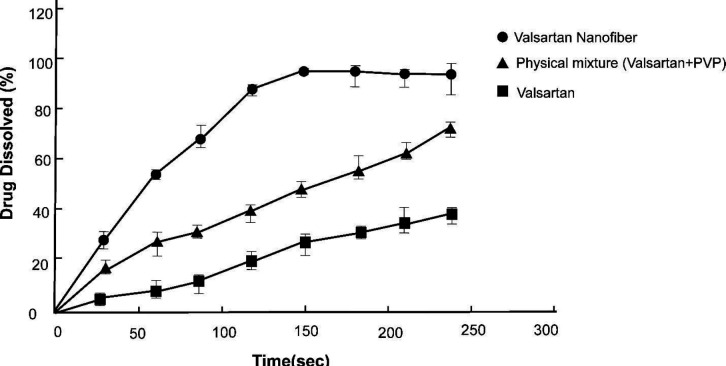
Diagram of valsartan release from the optimum nanofiber, dissolution rate of intact valsartan, and valsartan in the physical mixture with polymer at pH=6.8 (n=3)

## Conclusion

In this study, PVP electrospun nanofibers containing valsartan were produced with the aim of attaining orodispersible films (ODFs), and their properties were assessed. Firstly, PVPK90 was evaluated for the formation of nanofiber by variation of parameters. Thereafter, three parameters of polymer concentration, drug amount, and voltage were considered as variables and fifteen formulations were selected by Design-Expert software according to the mentioned variables. The required evaluations and tests were performed on these formulations. Finally, the formulation with 8% PVP and 18% valsartan, produced at 9 kV voltage, was selected as the optimum formulation. The SEM results showed that nanofibers were formed on the scale of nanometer and were homogeneous. The results of the tensile test validated the good flexibility of the selected formulation. The content test evaluation confirmed drug loading of above 90% in the selected nanofiber. Wetting and disintegration time was also short and acceptable in the selected formulation. The release profile of the drug implies rapid drug release that fits our research goals. Therefore, this kind of nanoformulation could be an effective platform for further *in vivo* and clinical studies to obtain more benefits from the vital antihypertensive medication, valsartan.

## Authors’ Contributions

AA Study conception and design; ZM and AA Data processing, collection, performing experiments; AA and MRA Analysis and interpretation of results; PI and NR Draft manuscript preparation, visualization; AA, MRA, NR, and PI Critical revision and editing of the article; PI, NR, ZM, MRA, and AA Final approval of the version to be published; AA Supervision, funding acquisition.

## Conflicts of Interest

The authors declare that there are no conflicts of interest.
